# Bodily Learning Through Creating Dance: Student Teachers' Experiences From Norwegian Physical Education Teacher Education

**DOI:** 10.3389/fspor.2021.758944

**Published:** 2021-10-29

**Authors:** Trine Ørbæk

**Affiliations:** Department of Educational Science, University of South-Eastern Norway, Kongsberg, Norway

**Keywords:** bodily learning, physical education teacher education, creative dance, micro-phenomenology, body memory

## Abstract

Creating dance in physical education teacher education (PETE) is described as a way of expressing subjective experiences based on movements that the students have already mastered or as a way of composing dance with set movements from various dance forms that are further explored through concepts such as time, space, power and flow. This article shows how 13 Norwegian student teachers experienced the creation of dance as part of their PETE. It explores the following questions: how do student teachers in PETE experience dance creation as an embodied process of exploring, transforming and creating movements, and how were these experiences facilitated by bodily learning that was initiated by body memories? The results show that creating dance is a intersubjective, intercorporeal and interaffective phenomenon where the sense-making process happens as both an individual and a joint process. This ongoing individual and participatory sense-making can further be understood as a reciprocal dependency between culture and self, where the students' bodily learning process evolves on an existential level over time. Through this process, the students develop a sense of confidence and trust in each other, which creates a feeling of belonging. The educational potential of bodily learning through creating dance can be seen in relation to the affordances the students perceive and utilize within their learning culture in PETE.

## Introduction

Creative dance as a dance form emerged in the beginning of the 20th century as a protest against established dance forms, such as classical ballet, that are based on established steps, movement patterns, as well as concepts (Daly, 1994). Creative dance focused on how “natural” and “everyday” movements could be included in dance, such as moving barefoot, running, rolling, limping, greeting and so on (Daly, 1994). Inspired by the dance artist Isadora Duncan (1877–1927) and her concept of *natural movements* (Daly, 1994), German *Ausdruckstanz* (expressive dance) and Dewey's child-centered pedagogical perspective, creative dance in an educational context developed into two different directions (Vertinsky, 2010; Ørbæk and Engelsrud, 2019). In USA, the physical educator at the University of Wisconsin and Trilling, Margaret N. H'Doubler (1889–1982) focused on how the creative process in dance starts by paying attention to one's own movement experiences, exploring these movements and creating further new movements alone or together with others (H'Doubler, 1998). H'Doubler (1998) emphasized the importance of that creating dance and dancing could be a personal, non-competitive, democratic activity in which students learn to express themselves, instead of focusing on movement mastery within established dance forms (H'Doubler, 1998). This experience-based approach to creating dance was further developed by Hawkins (1991), Fraleigh (2004), Hämäläinen (2006) and Sheets-Johnstone (2009), amongst others.

In Europe, the Slovakian-German dance teacher, choreographer and movement theorist Rudolf Laban (1878–1958) established the “Art of Movement Studio” in Manchester together with Lisa Ullmann. This studio became the main vehicle for Laban's modern dance and movement education in Europe (Laban, 1948). Based on movement analysis of observing industrial workers he developed the effort-theories as concepts for observing and analyzing movements (Laban and Lawrence, 1947). Laban (1966) describes these theories as a kind of grammar and syntax of the language and movement, dealing not only with the outer form of movement but also with the mental and emotional content. This was based on his belief that motion and emotion, form and content, body and mind, are inseparably united (Laban, 1966). In creative dance, his concepts of *body, effort, space* and *shape* are often used as starting points for exploring movements alone, and together with others (Laban, 1948)^1^.

These two directions indicate that teaching in creative dance can facilitate the students' natural and everyday movements and offer concepts to be used as starting points for movement explorations. In physical education teacher education (PETE) creative dance has been adopted in two different ways. First, teachers and teacher educators can understand creative dance as a way of expressing subjective experiences through creating dances based on natural and everyday movements the students already know (Chen and Cone, 2003; Minton, 2007; Cone, 2009; Torrents et al., 2012; Mattsson, 2016; Steinberg and Steinberg, 2016; Oppici et al., 2020; Payne and Costas, 2020; Lobel, 2021). Chappell (2007) labels this approach to creative dance, embodied way of knowing dance. Second, teachers and teacher educators can understand creative dance as a way of composing dances based on various dance forms with set movements. These can then be explored further through Laban's concepts of body, effort, space and shape (Keun and Hint, 2006; Morin, 2008; Rustad, 2013; Larsson and Karlefors, 2015; Cleland Donnelly and Millar, 2019; Rustad and Fjogstad Langnes, 2019; Velten Rothmund, 2019; Amado et al., 2020; Lara-Aparicio et al., 2021; Neville and Makopoulou, 2021). Chappel labels this approach to creative dance language-based way of knowing dance. Chappell (2007) continues that there is a tension between such an embodied and a language-based way of knowing in creating dance and a tension between incorporating and interrelating individual and collaborative creativity. Ørbæk and Engelsrud (2019) suggests that creative dance is about expressing subjective, bodily experience through dance that uses the body to express a dance that is created within different dance forms. This approach, thus, combines elements of both embodied and language-based approaches to creating dance.

Whereas, research in dance shows that creating dance can be experienced as an intersubjective, intercorporeal and interaffective phenomenon (Sklar, 2001; Anttila, 2006; Østern, 2009; Ravn, 2009; Foster, 2011; Reason and Reynolds, 2012; Rustad, 2013), there are only a few studies in a PETE-context that look at what students experience *bodily* through creating dance, as well as about the potential that creating dance may have for bodily learning and formation in a PETE context today. In this study, I will further explore how creating dance can be an embodied process of exploring, transforming and creating movements, and how this process can be facilitated through language-based learning employing concepts such as subjective, dyadic, intercorporeal and collective body memories.

In this article, I present a micro-phenomenological case study (Petitmengin et al., 2018; Varea et al., 2018) of the experiences of 13 Norwegian student teachers in creating dance as part of their PETE. Building on the studies presented above, I ask the following questions: how do student teachers in PETE experience dance creation as an embodied process of exploring, transforming and creating movements, and how were these experiences facilitated by bodily learning that was initiated by body memories? The first question will be presented and discussed together with the results of the study. The second question will be discussed in the conclusion. In exploring these questions, I draw on concepts such as *embodied inter/affectivity, body memory, social musicality* and *participatory sense-making*.

## Theoretical Perspective

In this study, I understand learning to be a socially interactive situation in which teachers and students are mutually involved in what the German philosopher Fuchs (2016) describes as *mutual incorporation*, where bodies create affect and are created in affective encounters. For Fuchs (2016), the emotional impression of being in a situation triggers a specific *bodily resonance*, which both creates affects and prepares the body for movement—-the *affective* and *emotive* component of emotions. He uses the concept of *embodied affectivity* to describe this sort of circular emotional interaction that the subject is part of with the environment (Fuchs, 2016, p. 197). Another form of circular emotional interaction occurs when subjects meet. Fuchs (2016, p. 198), derived from Merleau-Ponty's concepts intersubjectivity and intercorporeality, describes this as *embodied interaffectivity*. Such intercorporeal interaction takes place quickly, and people are not able to control it cognitively or rationally. In the classroom, the teacher and the students become parts of a dynamic sensorimotor and inter-affective system of this sort that connects their bodies by way of reciprocal movements and reactions, in what Fuchs (ibid.) describes as *interbodily resonance*. These bodily experiences form the existential basis of the class culture and the learning environment for each student and they include the teacher's and the students' body memories from previous learning situations.

According to Fuchs (2017), human bodies are to a large extent shaped by culture; this includes how bodies in a specific culture sit, stand, walk and dance. This intimate connection between culture and bodily learning is bound to a specific kind of memory—*body memory*—which Fuchs (2017, p. 333) describes as follows:

Through repeated and typical interactions with others an individual habitus is formed, and with it the norms and rules of culture are inscribed into the body, yet in such a way that the resulting memory corresponds to an embodied and implicit knowing *how*, not to a knowing or remembering *that*.

For Fuchs (2017, p. 335), our embodied and implicit knowing “is not made accessible to us in retrospect, but is re-enacted through the practices of everyday life.” He defines the entirety of established dispositions and skills as body memories that become current through the medium of the lived body without the need to remember earlier situations (Merleau-Ponty, 2012). For Fuchs (2017, p. 335), body memory comprises “all those habits, manners, skills and practices that are performed prereflectively” and includes “habitual bodily interactions with others.”

Fuchs (2017, p. 340) states that a great part of our *body memory* has been passed from one generation to the next through performative practices and specifically socialized bodies. Instead of seeing bodies as symbols of cultural ideologies, Fuchs (2017) focuses on the pre-reflective layers of experience that enable an examination of expressive qualities of lived body unfold, for example, in the cultural practices of dancing. Against this background, Fuchs (2016) argues that body memory may serve as the mediator between the lived body and the history of culture. These interbodily interactions develop *collective body memory* that, according to Fuchs (2017, p. 341):

is based, on the one hand, on acquired dispositions of the individuals; on the other hand, it is actualized only through the interactions of the group as a whole. […] [I]t is also particularly suited to carry the identity of the group and make it tangible for its members.

Collective body memory is a sphere of prereflective mutual bodily attunement that enables the formation and tradition of collective patterns of interaction (Fuchs, 2017). Repeated patterns of interaction create affective-interactive schemas that become familiar and result in a prereflective, practical knowledge of how to get along with others. This *social musicality* (Fuchs, 2016, p. 196) is developed from the time we are born and is like “a practical sense, a musicality for the rhythms, dynamics and patterns of interactions with others [where] intermodal kinematics and bodily resonance are key to attuning and sharing each other's affects within the primary dyad” (Fuchs, 2016, p. 205). From this perspective, bodily learning processes include enacting, exploring and creating new intercorporeal interactions according to the patterns extracted from our previous bodily experiences (Fuchs, 2017). According to Fuchs (2017, p. 338), all our “interactions are based on such integrated bodily, emotional and behavioral dispositions, which have become second nature, like walking. They are now part of one's embodied personality structure.” These connections mean that the sense-making of interactors acquires coherence through their interaction, not just in their physical manifestation but also in their significance De Jaegher and Di Paolo (2007). This is what De Jaegher and Di Paolo (2007, p. 489) call *participatory sense-making*, which can be understood as on the spectrum of participation:

At one end of the scale, sense-making remains largely an individual activity that is at most modulated by the existence of coordination in interaction. At the other end, where participation is highest, we fully and directly participate in a joint process of sense-making and the whole sense-making activity becomes a shared one. (De Jaegher and Di Paolo, 2007, p. 496).

In line with Fuchs (2017, p. 339), such a sense-making process is achieved through prereflective bodily attunement (De Jaegher and Di Paolo, 2007).

According to Fuchs (2016), close relationships between two persons can create *dyadic body memories* that are memories of how the two move together in different situations. It manifests itself in shared patterns of interaction, which are actualised every time the two persons meet again. The respective intercorporeal memories of the partners unite to form a joint procedural field that suggests and preordains certain typical postures, interactions and interaffective experiences. Both body schemas are attuned to each other through resonant kinaesthetic patterns and thus interenact their shared history: rituals of welcoming, joint repertoires of gestures, postures and movements, which “fall into” the “presence of the other, as a kind of unintentional entrainment” (Fuchs, 2017, p. 339). In such a mutual incorporation, the interactors will experience a specific feeling of being bodily connected with the others, as I will show later when the students dare to create dance when they work together with a student peer in whom they have confidence.

## Methodology and Methods

On the basis of previous research on creative dance, the above concepts and theoretical perspectives, I have designed a micro-phenomenological case study (Petitmengin et al., 2018; Varea et al., 2018) where I have re-analyzed the student teachers' reflection notes and interviews that I explored as part of my PhD dissertation (Ørbæk, 2018). The research questions that guides this study are: How do student teachers in PETE experience the creation of dance? How can these experiences contribute to—and illuminate—creating dance as a bodily learning process in PETE?

### Research Design

Micro-phenomenological case studies focuses on exploring emotional and bodily dimensions of our lived experience and describe them accurately and reliably (Petitmengin et al., 2018; Varea et al., 2018). In this study, I investigate my own student teachers' experiences when create dance as an obligatory part of their 10 ECT dance course in their PETE. In the classes, the students created dances both from their own movement experiences and from using the concepts body, effort, shape and space (Laban and Lawrence, 1947; H'Doubler, 1998). Research in own teaching practice in higher education belongs to the scholarship of teaching and learning (SoTL) research tradition (Boyer, 1990). Research within the scholarship of teaching focuses on teaching practices in higher education and quality of teaching, and research within the scholarship of learning, where this study is located, includes research on student learning (Booth and Woollacott, 2015). I also locate this study within the emerging field of performative research within higher education, which focuses on how relational, affective and emotive experiences are included in learning and teaching in teacher education (Østern and Engelsrud, 2014; Bae, 2016; Østern and Nødtvedt Knudsen, 2019; Østern et al., 2019, 2021; Ørbæk, 2021).

### Data Material

The data material stems from the student teachers' PETE from August 2011 to December 2012 and from my PhD dissertation (Ørbæk, 2018), approved by the Norwegian Centre for Research Data. The informants are students who began their PETE in the autumn of 2011. The selection criteria included: (a) being a student teacher in physical education and (b) either being confident in various dance forms and looking forward to creating and teaching dance in PE or else lacking dance experience and being uncertain about creating and teaching dance in PE. I have given the students the following fictive names: Anders (male), Knut (male), Charlotte (female) and Ella (female). These four students had prior experience of learning various dance forms, such as salsa, swing, folk dance and so on. Caroline (female), Gina (female), Jens (male), Mons (male), Thomas (male), Vidar (male), Sigurd (male), Henrik (male), and Daniel (male) lacked dance experience of various dance forms. None of these 13 student teachers, who were between 21 and 27 years of age, had experience of creating dance of their own.

The data material consists of two transcribed individual interviews (I1, I2) and two sets of obligatory reflection notes that were evaluated and anonymized by a colleague (R1, R2). The reflection notes were written in response to an obligatory task that all students completed after the first and their 2nd years of their PETE study. The notes include descriptions and reflections on a dilemma the students experienced when creating dance. I asked the students to think of a situation in which they had felt uncertain about how to act. They were allowed to refer to similar situations in books they had read and in movies and conversation with others, at various places and during sport experiences and so on. They were to write from a first-person perspective and they were encouraged to include descriptions of their feelings and thoughts about their experience of creating dance alone and together with other students. The content in these reflection notes informed the questions in the interview guide. The first interview was conducted immediately after the students' practicum period in the 2nd year, and the second interview took place after the students had finished their final course and exam in creative dance, in December 2012/January 2013 (Kvale and Brinkmann, 2009). This final interview was important in order to allow for the students to include both challenging and good experiences without experiencing ties to me as a teacher.

In order to develop a more nuanced understanding of what the students had done, thought and felt when creating dance, I conducted what Petitmengin et al. (2018) describe as a micro-phenomenological interview. A micro-phenomenological interview produces descriptions of actions and is used mainly in the field of education to gather precise descriptions of experiences of specific situations from a first-person perspective (Petitmengin et al., 2018). Micro-phenomenological interviews provide an opportunity to produce data material that also reveals emotional and bodily dimensions of an individual's experiences. The focus in the interview situation with the students was on using what they had written and talked about as a method of bringing to life the situation that I wanted to know more about. I then let the students talk about what they thought, did and felt in their bodies before, during and after this situation. The aim was to elicit their reflections and meta-reflections on their kinaesthetic, affective, interaffective and emotive experiences (Fuchs, 2016). Finding words for the students' bodily and interbodily experiences when creating dance was challenging both for the students and for me in terms of trying to grasp their experience in words, and sometimes it was not easy to know how to understand what they were trying to say. Eliciting the students' experiences became a round dance between my bodily impression of what they wanted to say, what they put into words, and the words that I suggested they might use to describe their experiences. In such an embodied interaffective situation, as Fuchs (2016) calls it, the students and I developed words, sentences and descriptions that could illuminate the experiences they had created. It was possible to achieve such an embodied development of language because I already had a close and trusting relationship with the students. Being able to include reflections on kinaesthetic, affective, interaffective and emotive experiences from my own experience of creating dance as a choreographer helped to strengthen what Bresler (2006) describes as the significant empathic listening in qualitative interviews. Researching with such proximity and inside perspective to the student teachers' experiences of creating dance in my own teaching practice is a form of research which, according to Østern (2017), is oriented toward meaning, interpretation, understanding and change in teaching practices.

### Analytical Approach

When I started re-reading through the data material I had analyzed as part of my PhD (Ørbæk, 2018), I became aware that the students' experiences centered on detailed descriptions of eight specific situations from various teaching situations. These situations were common to all students, who referred to them both in their reflection notes and in the individual interviews. I began the micro-phenomenological analytical approach (Petitmengin et al., 2018; Varea et al., 2018) by organizing the descriptions of each student's singular experience of the eight situations. Then I analyzed generic and specific structures of the students' experiences of the same situation: what Petitmengin et al. (2018) refer to as the synchronic structure of the experience. In this study, the process included eliciting descriptions of the student teachers' affective and interaffective experiences and sense-making when creating dance as well as their reflections on these experiences. In order to highlight the temporal development of the students' experiences—the diachronic structure of the experiences, which according to Petitmengin et al. (2018), characterizes the presentation of micro-phenomenological analyses—I have chosen to present the eight units of meaning (descriptemes) in chronological order.

## Results

The first descripteme is about the students' experiences of “dreading the lessons.” This is followed by the descripteme of “creating dance based on own movement experiences,” which shows the importance of using movements the students already know and like. The third descripteme, “anxiety about not fitting in,” relates to how the students choose to move in similar ways to the others in order to make the others like them. In the fourth descripteme, “the fear of, and the desire to, express emotions,” I show how the students react when they are given the task of moving as they feel. The fifth descripteme, “confidence to create dance,” illuminates how dancing together in pairs opened up new ways of exploring movement in dance. The sixth descripteme, “sharing and adjusting to other's movement experiences,” focuses on how the students experienced the creation of dance in groups of four to six fellow students. The seventh descripteme, “exploring own movement experiences, movement opportunities and movement interests,” shows how the students experienced creating dance alone, based on their own movement opportunities and movement interests. The last descripteme, “new ways of moving and understanding the creation of dance,” shows how students talk about the experience of exploring new ways of moving when creating dance.

Each descripteme follows the same structure. First, the students' experiences of the situation are presented from a first-person perspective, in language that is close to that of the students' oral and written descriptions, as expressed in the empirical data. The focus is on showing how the students thought, what they did and how they felt in the situation. I then describe these experiences further on the basis of the chosen theoretical perspectives and concepts.

### Dreading the Lessons

Henrik (HR1) is sitting in the wardrobe. He has to change clothes before dance class. When he woke up this morning, the first thing he thought was, “Oh no, dance AGAIN!” Dancing is one of the worst things he knows. He wonders if the lessons today will be the same as for teaching aerobics. He “hates aerobics.” Even though he had done his best, he failed the course. He is afraid that the same thing will happen in dance. He puts on his shorts, is shaky and nervous. Finally, he gets his shoes on. He takes a deep breath and thinks: “I guess I can just do my best one more time. I cannot do something better than that.” The walk from the locker room to the dance hall seems long, much longer than it has before, such as when football was on the schedule. Henrik hears the sound of the music from the distant room. He just wants to turn around, but he gathers his courage and continues to walk “the long corridor.” When he finally arrives, after “getting his head up” and motivating himself, he opens the door and is greeted with a smile from the teacher. The smile helps him find a place at the back of the room. The “worst” thing he knows is doing dance moves while someone else is watching. Therefore, he will stand at the back. He gets into the circle for roll call at the beginning of the class. He listens to today's topic and feels the doubt occupy his body.

Gina (GR1) sits in the circle and becomes aware of the “hard floor.” She is unwell and thinks that dancing is not something for her “uncoordinated body.” She is wondering what's going to happen. Anders (AR1) has danced many types of dance before, such as folk dance, salsa and street dance. His experience of dance classes is that the quality of the lesson depends on who is teaching. He is excited about how the lessons in dance will be carried out. Thomas (TR1) comes straight from the chiropractor with a rib lock and has pain in his body. Jens (JR1) is full of expectations, tensions, nervousness and joy. “I'm not a dancer,” he thinks, “but I'm a little creative.” He longs to be able to dance with “well-coordinated, trained movements.” But he does not think he will achieve this, nor does he believe that he can contribute any good dance moves this time. He has low expectations of his own dance performance. When he compares himself to others, the word “dance” fills him with a feeling of inadequacy. It gives him a “bad feeling.” On the other hand, he wants to “have fun, be safe, and use his creativity for something fun, like when [he] plays football.” He wants to create something with people he is confident in. He wants to challenge himself and use his “resources.” He believes the limitation in creating dance lies in his lack of belief in himself “when it comes to unfolding to music” in a setting where others may have an opinion about or see and experience what he presents. Now he is pleased to have the opportunity to “use all his senses and skills in a creative way,” but he “feels a nervousness about the unknown.” He is afraid to go into something when he does not know what it is about or what is.

The students' previous bodily experiences of dance and creating dance in physical education become visible in the data material. The students bring with them, as Mattsson (2016) and Rustad and Fjogstad Langnes (2019) have pointed out, an understanding that dance is about performing movements within various dance forms, such as aerobics and folk dance. Such an understanding of dance, as a step, is described by Velten Rothmund (2019, p. 17) as “dance technique as something set,” and in order to create dance it is important to first learn the predetermined movements that characterize the dance form. When the students were about to start creating dance, the preconception became apparent of performing movements within various dance forms, such as movements within the dance forms of hip hop, salsa, disco, swing or folk dance. Mattsson (2016) writes that ideas and expectations that dancing involves knowing certain steps to music, couple dancing and so on are something that teachers in physical education and student teachers may have a collective body memory of Fuchs (2017). Seeing dance as mastering movements is a habitus that, according to Mattsson (2016), is easy to maintain, both for students and for teachers in a PE context.

The students also remember situations where they have danced different forms of dance on the basis of predetermined steps. Their intercorporeal and collective body memories, to borrow concepts from Fuchs (2017), are activated when they think about dancing. The thought of experiencing oneself as inadequate emerges, which activates unrest and reluctance. Previous bodily experiences from dancing contribute to the students' bodily experience of the hall, the “hard” floor and the corridor being “long.” They become especially aware of their own movement limitations, such as pain in the body and what they think of as a lack of skills in various dance movements. Reluctance and fear of what others will think about how they move also emerge. In fact, what Fuchs (2016) calls *mutual incorporation* begins even before the students have begun to create dance.

At the same time, themes emerge, such as “using creativity for something fun,” “having fun,” “creating something together with fellow students,” and “challenging oneself.” Despite the fact that students understand creative dance and dance technique as movement performance within different dance forms (Velten Rothmund, 2019), there is also curiosity about whether creating dance may be more than performing dance steps within different dance forms.

In connection with the next theme, “creating dance based on own movement experiences,” I will share how the students experienced the creation of dance based on movements they already knew and liked. As a result of my observations in previous classes with these students, I had become aware of their shared interest in doing tricks with a football. In order to make them start to create dance on the basis of their own movement experiences, I created a task where the students, in small groups of four to six students, would do tricks with a football without any football and imagining a football going back and forth within the group.

### Creating Dance Based on Own Movement Experiences

Gina (GR1) begins to wonder if she has come to the “wrong class.” Can this be right? Football in dance class? This isn't dance, is it? Doing tricks with a football? Or will she get to dance and create dance out of doing tricks with a football? Maybe. Her feelings go from “I cannot do this” to “Maybe I can do this anyway.” She is ready. Everyone around her is moving at full speed. Football. “Fantastic! But, where are the balls?” The students take a quick look around but soon take in the point of ball tricks without a ball. Imaginary balls “fly around” the room, and Gina feels that the ball is being played “back and forth between the eight legs” that are in the ring she is part of. The ball moves from toe to heel with the “most amazing kicks and tricks” being performed. The rhythmic and evocative music in the background accompanies the football movements and transforms “the most solid football trick match” into “a dance from another universe.” Gina no longer feels unwell. The feeling of powerlessness has turned into a feeling of joy and mastery. She gets to dance. In her own way.

Henrik (HR1) has joined another group of football players. They quickly start tricking. It is fun to move around with an imaginary football. “I hope all the classes are like this!” Henrik and the others in the group do tricks with the invisible ball “over the neck,” they put it “dead on the ankle,” make a “round the ground,” put the ball “dead on the forehead” and play with the ball as if they were “standing on the football field with a real ball.” The sound of the music “thumps” in the background. Finally, the music “beats” in time with the students' hits on the ball. For the first time, Henrik gets a “little taste of mastery” when it comes to dancing.

Jens (JR1) says that his desire to dance rises up “like “Himalayas!” when he can start from movements he is already well-acquainted with. Although it seemed “very special” to do tricks without a ball, it was easy to imagine that he had a ball. Jens imagines that he goes out on the artificial turf field and starts doing tricks with the ball. It's easy. He does tricks better than ever. The ball goes from foot to foot, steady and nice. The “touches” are balanced and rhythmic. He does tricks with an invisible ball “which suddenly becomes visible” to him. He remembers how as a child he stood out in the garden and did tricks day in and day out. He dreams of going on boat trips where he does a trick with the football on “three square meters of boulders.” Doing tricks with a ball has always had intrinsic value and been a joy for him. He feels very close to this joy now. Creating dance based on football trick movements gives him a “delicious feeling of mastery,” an “absurd feeling without roots in reality.”

The theme of “creating dance based on own movement experiences” contrasts with the theme of “dreading the lessons.” This may seem like a somewhat quick transition from one to the other here, which may have to do with the fact that I made an effort for the students to experience it this way. This task was based on research by Neal (1981), Chen (2000), Engel (2004), and Nilges (2004) that shows that students in PETE like dancing if the movements are based on movements they already know and like. Doing tricks with a football without an actual football activates the students' body memories of doing tricks with a football (Fuchs, 2017) and the students manage to perform specialized trick movements, such as putting the ball “dead on the ankle” or “around the world.” For some students, such as Jens, the trick movements also evoke emotional memories, which shows that meaningful and unique experiences, or meaningful experiences, according to De Jaegher and Di Paolo (2007), can be experienced again by moving in similar ways, even in new contexts.

The students' experience of creating dance based on their body memory of doing tricks with a ball emerges as a circular round dance between tricks with a football and the experience of tricks with a football, which gives rise to what Fuchs (2016) describes as a bodily resonance, which made the students feel good while dancing. Expressions such as “fantastic kicks and tricks,” “we played with the ball,” “the trick better than ever,” “a dance performance from another universe,” “a feeling of mastery when it comes to dance,” and an “absurd feeling without roots in reality” show that the students explored new ways of moving in dance. The coordinated rhythmic movement to the music, and the shared feelings it evokes, played a crucial role in creating dance based on the students' body memories of doing tricks with a football. These new dance experiences also developed a feeling of mastery and well-being when dancing. In light of the concept of *social musicality* (Fuchs, 2016), the students acquired competence in creating dance when they managed—together—to develop their previous body memories into a dance. By using Fuchs' (2017) concept of operative we-intentionality, the students had the experience of connecting to one another's bodies through skilful interaction, achieved through their bodily attunement to one another. For the students, this way of creating dance opened up a procedural field of possibilities and an affordance for creating dance.

However, the students found that there was not always room to use their body memories as a starting point for creating dance. In the next theme, I will show the students experience of creating dance on the basis of their own way of moving to music mixed with movements they imitate from other students moving in the same room.

### Anxiety About Not Fitting in

The students begin to walk around the room. They notice each other. Their walking develops rapidly into hip-hop movements and movements from disco dance, swing and various folk dances. Those with experience of various dance forms are in the lead and suggest movements that the other students try to imitate. In other words, the movements of those who “can dance” have status and work in the bodies of fellow students, who are “forced” to “copy” (AI1) the movements. Mastering set dance movements within various dance forms gives status, but Anders (AI1) finds that there is still room for moving “a little differently” than the student he is trying to “copy.” According to Anders, it is not possible to perform a movement in exactly the same way because he must “interpret” the movement with his body.

For the students, it is important that the movements of their fellow students whom they choose to imitate are similar, and that the difference between their “own” (AI2) and “other people's” (AI2) movements do not differ too much. If the gap between the way the fellow student moves and the way the student performs those moves is too big, it entails a risk of not fitting in with the other students. When Anders tries to “copy” (AI2) the way his fellow students move, he wants to “fit into the crowd” and thus ensure that he is included in the class community. He thinks that many of the other students feel the same way. If Anders had explored the movements that he “want[ed] to explore,” he assumes that the others in the class would not have been able to “identify” with him, because he likes to explore different dance forms that he thinks his fellow students would see as “different” and “rare.” He wanted to avoid that. Anders thinks that if he chooses movements that are similar to the movements of others, the others will not be able to judge him, because then he can say: “Yes, but you did almost the same thing yourself. What is the difference?” Creating dance according to such a norm of experienced movement means that the students compare movements with each other and seek social affiliation by moving in similar ways. At the same time, they are anxious about feeling “excluded” from the others in the class if they are not allowed to move in similar ways.

When the students began to move in the room, their preconceptions and collective body memories of how to move in a dance class were activated (Fuchs, 2017). This shows that the students brought with them culturally established norms of how to move in a dance class, something that Mattsson (2016) also points out. The experiences the students had tricking the football in class were lost in the task of walking, and the expectations in the dance class had related to social comparison, which here emerges in the comparison of movements. Through the concept of *habitus* (Fuchs, 2016), the movements from different dance forms were the most valued practice in creating dance, and everyday movements and sports movements were experiences that were “out of place” in a dance class. The students who had already mastered the set dance movements set the scene for the movements that were allowed—and those that were not. However, the students, both with and without experience of different forms of dance, were concerned with fitting in with the class community and adapting their movements to one another. By influencing and being influenced by one another's movements, the students created a movement norm in dance for this class. Expressions such as feeling “stupid” and being afraid of being “judged” or not “being liked” show that dance and creating dance, based on such a class norm, is part of, and perpetuates, feelings of inadequacy and uneasiness about not fitting in with the social community of the class. This is a cultural aspect of dance which contributes to a student to “feel[ing] stupid” and thinking “I cannot dance,” something that Rustad (2013) also shows us.

This ongoing intercorporeal interaction between the students both limits and promotes the movements that the students choose to include in their dances. In order to reduce the risk of being excluded and to increase the likelihood of being liked by the others, they adapt to one another's movements, trying to *mutually incorporate* each other (Fuchs and De Jaegher, 2009). According to H'Doubler (1998), the need to be equal, realized by imitating each other's movements, can be understood as a need to fit in with the class community: “[W]e dance as others are dancing—afraid to be ourselves if to be ourselves means to be different. We fear what others might say (…). We feel safe in conforming, in being like others” (H'Doubler, 1998, p. 28). Although students in other movement situations in PETE may experience confidence in the way they move, creating dance is a new situation for most of the students. The need to fit in was more important than exploring individual movement interests. Incorporating one another's movements within the limit of the class's movement norm is difficult for many students, especially those who lack experience of creating dance. This became even more apparent when the students were to move the way they felt when they were listening to a song I had chosen for the class.

### The Fear of, and the Desire to, Express Emotions

Caroline (CHI2) stands at the back of the room in a dense cluster with her fellow students. She stares at the floor, thinking about how she can solve the task of moving the way she feels. She thinks the task is “too broad.” There are far too many choices for how to move. Therefore, she stands completely still, like the others in the class. Caroline thinks that the teacher is completely “out of her mind.” Several of the others think so too. Caroline feels that she and her fellow students are in shock. “Stiff as sticks,” they stand close together. She hears the teacher ask them: “What just happened? Why did you cluster together so far away from me?” No one answers. They do not know what happened. All they know is that it feels important for them to stand close together. It makes them safer.

The students begin to look at each other. Their shared wonder is felt in “the air” around them: “Does the teacher really mean that we should just go out and do something on the floor here—-now?” (CI2). None of them are looking at each other. Their eyes are still focused on the floor. Caroline has “lots of different feelings inside her,” but she finds it difficult to find the “right” movement to express the “exact feeling” she wants to express. Creating movements and then “adding a feeling” afterwards feels “wrong” to her. Caroline thinks it is easier to create dance when she first explores movements she likes to make, because these movements do “something right” to her. They give her a good feeling. While standing still in the cluster, she feels that the music “takes hold” of her, and she wants to “do something” in relation to the music, based on the movements she likes to make. But she does not dare. She has “[a lot of] difficulty in doing such things” because she is not “used to” creating dance. Caroline says: “I do not just want to stand there and express myself if I am having a bad day, or if I am sorry somehow and make movements that express that I am sorry!” She thinks about what the others might think of what she is doing if she moves the way she feels. Daniel's challenge is that he does not dare to “be himself 100 percent” (DR2) in a classroom context. Although he feels that he has the freedom to make exactly the movements he wants, the “little barrier” is there. The idea of “failing” and “fooling around” in the other's eyes stops him from moving. The thought of going out on the floor and starting to move based on how he feels is scary. It feels safer to stand still—like all the others.

Gathering in a cluster, as the students did in this task, can be seen in the light of Fuchs' (2016) concept of emotion. For Fuchs (2016), emotions are the bodily expression of affective and emotive experiences in a given situation. When the students gathered in a cluster, it can be seen that the students had actually performed the task they had been given. By quickly gathering in a group, they created a dance that best expressed how they felt in the situation. The uncertainty about how they would manage to express emotions created a bodily disturbance that quickly brought the students together in order to feel safe. According to Fuchs (2016), we can know each other's bodies in a situation, and even experience being known by other bodies, and experience someone else experiencing us bodily. In this mutual incorporation of one another, the students no longer experience themselves as clearly separate bodies but as extended, and they connect through a jointly felt sense of being insecure and afraid. In this shared intercorporeal interaction, the students perform coordinated interactions while also constituting a meaning of their interaction that makes them feel safe in the challenging situation. Hence, their interbodily resonance enhances the sense of class community (Froese and Fuchs, 2012). The idea that arises is that expressing “oneself” through an emotion is not something the students want to do, but that creating movements that express the experience of listening to music is something they do want to achieve. In accordance with concepts from Fuchs (2016), the students desire to “dare” to express their affective and emotive experience of music through their own movement experiences. What stops them from moving is the inhibitory norm of movements in dance established in the class.

According to H'Doubler (1998), individuals may feel confident that they have the “freedom and opportunity” to move as they wish, but they are too insecure “within [them]selves as individuals” to do so (H'Doubler, 1998, p. 28). Although the students are confident that they can move in diverse ways, they do not feel that there is room to express “themselves” in the classroom context. The movement norm established in the classroom creates a fear of what the consequence of “fooling oneself” might be. According to H'Doubler (1998), the need to appear to be like everyone else is a general need in humans, but this attitude “quells the spirit of inquiry and the impulse to create. The result is a rigid living that breeds distrust and intolerance of other ways of life” (H'Doubler, 1998, p. 28). H'Doubler (1998) uses the terms “distrust” and “intolerance of other ways of life,” which may characterize the environment experienced by the students in this situation. The students share a collective body memory in which the class has a low level of tolerance for other ways of moving in dance than those established as the class's movement norm in the dance lessons. When the students were given the task of creating dance that expressed emotions, a “barrier” arose inside them. They did not want to express emotions within the established norm of movement, nor did they dare to move beyond the established norm of movement by expressing feeling through movements they wanted to explore.

I had observed that each time I gave them a task, they paired up, even when they were supposed to work individually; in order to find other ways of creating dance based on the students' bodily experiences and movement interests, I now gave them the task of creating dance together in pairs. Under the next theme, I show the students' experiences of creating dance when they worked with a fellow student whom they were confident in.

### Confidence to Create Dance

Charlotte (CHI2) quickly joins someone who she knows well in the class. She sees that many of the others do the same. In contrast with being afraid of what the others in the class may think or feel about how she moves in dance, she finds that it is “completely natural” to move with a fellow student whom she is confident in. Now, she dares to explore a wider range of movements, because she knows the other student does not think she is “stupid anyway.” Moving with a person she knows well opens up the way for other movements, in contrast with when she is creating dance based on the class's movement norm. Now she includes, as do most of the students in the class, movements that she has performed only at home in the living room, on the dance floor or in the city and/or movements she has experienced in everyday situations, such as getting up or lying down in bed. For Daniel, this kind of movement exploration has to do with “[being able] to take the dance steps out of the living room” and “daring and challenging oneself” (DR2).

When the students created dances with a fellow student they were confident in, they dared to include everyday movements in their dances. This approach encouraged the students to create dances also with students they hardly knew. Eventually, through various movement tasks, most of the students create dances with each other in pairs. According to Charlotte (CHI2), creating dance together in pairs has made the students “very confident” in each other, and she finds that nothing anyone says or does is “wrong” or “stupid.” She thinks that most of the people in the class feel “so confident in the whole class” and in what creative dance is and that everyone can “come up with something” when they create dance together. Gradually, as the students become confident in each other through creating dances, new movements are also accepted. Charlotte says that the students have changed from trying to fit the class norm to just “danc[ing] like nobody is watching them” and just having “fun together.” They no longer need to be careful about being “similar to each other,” but rather put more energy into exploring and trying out new movements based on their movement experiences and movement interests. The previous understanding of creating dance that dominated their vocabulary and mindset as “wrong” or “stupid” retreats into the background. The focus is now on how to include their own movement experiences in the dances—something all the students manage to do and like doing.

According to Fuchs (2017), close friends develop a special form of body memory – a dyadic body memory – which is activated every time they meet, even after many years. When students who have confidence in each other start to create dance, their previous shared patterns of interaction appear in how they move, speak and feel in their present ongoing intercorporeal interaction. According to Fuchs (2016), when they start to create dance, they use their dyadic body memory to explore their shared movement experiences, which become the starting point for their creative process. In this process, their bodies “literally extend and connect with each other to form an overarching dynamic process […]. [T]heir interaction has gained an autonomy of its own” (Fuchs, 2016, p. 205). These dyadic body memories are not about what *I* remember, but about what *we* remember. Through the students' trustful *we*-relationship, they dare to further explore their own and their shared movement experience, which opens up the way for new movement experiences and the development of new dyadic body memories (Fuchs, 2017).

Creating dance based on dyadic body memories with another student leads to a willingness to explore the possibilities of movement with other students in the class. This shows how the students, through creating dance in pairs, also develop a trusting learning environment (Rustad and Fjogstad Langnes, 2019), which Nilges (2004) describes as the intersubjective dimension of creating dance. Through the lens of Fuchs (2016), the students' ongoing embodied affective and interaffective processes when creating dance together in pairs open the way for sharing and exploring movement with each other, creating a sense of belonging to each other – as Rustad and Fjogstad Langnes (2019) and Amado et al. (2020) also show. This process is characterized by curiosity, courage and respect for the diversity of movements the students bring into the creative process. Including movements from everyday life in their dances contrasts with the established movement norm that limits their movement explorations, as I have shown under previous themes.

Even if after this task the students thought that creating dance was “easy” and something that “everyone” could do and liked to do, their experience of creating dance in groups of four to six students provided other experiences.

### Sharing and Adjusting to Others' Movement Experiences

Vidar (VI2) and the four other boys in his group stand in a circle and discuss how to solve the group dance task. They are interested in moving in familiar ways and decide to create a dance about driving to the store to shop for food. According to Vidar, it is easier for him to create dance if he “has a story” that he can tell through movements. A story provides a framework he can try to “fit into” and gives him guidance for how the dance “should look when it is finished.” Vidar thinks it is “very easy” to “find” movements for the dance if the story is based on everyday life activities such as driving to the supermarket, which is suitable for this task because he has opened the car door and sat in the car “a thousand times” before. He knows the moves, and it feels “very natural” to perform them. Daniel (DR2) thinks that creating dance with others is the “most fun” because of the good “relationship” he has with the others in the group. They “cooperate” well during the process and they feel “proud” about performing the dance they have created. However, he thinks it is “strange” that he feels it is “fun” to create a group dance, and he wonders why he wants to do “more of this.” The experience of creating dance with the others in his group gives him a “good feeling” and a feeling of “succeeding – no matter what!”

Mons (MOR2) also likes to create dance with the others in his group, but he encounters a “dilemma” when his group is about to combine the various movements chosen by each student from their own movement experiences. What makes it difficult for Mons is that the movement he has chosen is a little “different” than the others' movements and therefore he has to adjust his movement to fit in with “the others' movements.” His dilemma is how he can adjust his movement without interrupting the rhythm he has chosen for his movement and without getting the group “out of balance.” After discussing this for a while, the group chooses to “redo a bit” of his movement in order to obtain a better “flow,” after which it becomes “easier to perform.” According to Mons, it is “common that something has to be adjusted [in order] for the whole to be as good as possible.” However, some of the students find this movement adjustment limiting. Ella (EI2) says she becomes more “blocked” and “locked” when she creates dance in a larger group, and she adapts more to how the others in the group move. Ella thinks this “interaction” is “very exciting” but also limiting, because she cannot move based on what she “comes up with” and without having to relate to anyone else. According to Thomas (TI2), creating dance with four to five other students is more about “collaboration” than about mastering a dance. Challenges to this collaboration arise, however, when there is a “strong person in the group,” which limits the other students' “creativity.” If this “leader” is also going to decide whose movements will – or will not – be included in the final dance, then the other students would prefer to create dance with others in smaller groups or alone.

In line with Chen and Cone (2003) and Cone (2009), these experiences show that the students were able to create dance if they were allowed to take their starting point in their own ideas and life experiences. When the students used “stories” and/or everyday situations, everyone in the group had experience to draw on for the structure and content of their dances; their collective body memories of driving a car, shopping and getting in and out of the car were quickly activated and opened the way for further exploration. According to Fuchs (2017), even if we share body memories of similar situations, our second-nature movements remain unique to each individual. This means that when students are to create dance based on body memories from everyday life, each student has their own unique way of driving, shopping and getting in and out of a car. When the group managed to include these unique movements from each of the students, the group members also managed to mutually incorporate one another, experiencing – through their bodies – the ways that the others had experienced the same situations. Within this overarching dynamic process they no longer experience one another as clearly separate from but as part of a whole. Such *wholeness*, as H'Doubler (1998, p. 140) calls it, is created by the students influencing and being influenced by one another's movements, where the movements from their own movement experiences are adapted to the movements of others. In this intercorporeal interaction, students are able to share and develop their own and one another's movement experiences. At the same time, they construct common ground where the process of creating dance is characterized by the unique movements and co-determination of the students. If the movement experiences of all of the students are included in the dance, they experience a sense of belonging to the group. This contributes to the fact that creating dance is something they achieve, something that gives them a feeling of happiness, and something that motivates them to continue creating dance together. In this way, creating dance makes the students become attuned to one another and aware of their intercorporeal experiences, and it develops their social musicality (Fuchs, 2016). However, such participatory sense-making (De Jaegher and Di Paolo, 2007) does not occur if a movement becomes too difficult to adapt to the movements of the others, if the students do not dare to move as they want or if one member of the group takes on the role of a leader who chooses which movements to include or exclude. Exclusion of the movements of some students made those students exclude themselves from the group and want to explore other ways of creating dance, mostly alone. According to Fuchs (2017), this may be seen as a shift from being included in joint dancing with the others in the group to being excluded from the group's movement patterns and thus starting to explore creating dance based on their own individual body memories.

### Exploring One's Own Movement Experiences, Movement Opportunities and Movement Interests

Ella (EI1) begins to move. When she creates dance alone, she activates movements “from the past,” but she does not remember the contexts in which those movements arose. She thinks the movements “lie a little inside” her. Since Ella has danced before, she thinks she probably has the dance movements a bit “in her.” When she creates dance, she uses the movements she “has” from a “bodily experience.” However, it is her experience that these movements have become a part of “her” and are something she can use when she creates dance alone. Vidar (VI1) also finds that the movements he makes when he creates dance alone are not “necessarily” new. He believes that everything he does is a “result” of the “influences” he has experienced before. When he moves, the movements do not come “completely out of the blue,” but they are not quite the same as before. When he makes the familiar movements in new “contexts,” such as in a classroom situation, he “transforms” them for the situation he is in. In order to transform these movements, Thomas (TI2) finds that it is necessary to think “outside the box” or to create a “new box.” For Thomas, the box is synonymous with “standard movements,” movements that he has seen in films and which he “recognizes immediately.” When he creates dance alone, he wants to use movements from the box, but he also wants to “mix” the “standard movements” with his own movements. In mixing known and unknown movements, he has the experience of “making something new.”

Creating dance by mixing familiar with unfamiliar movements provides two different ways of experiencing the body. When students move with predetermined dance movements within various dance forms, they experience their bodies from the “outside.” When they move based on their own movement experiences, they experience being in the movement with their whole bodily being. When Anders (AI2) is exploring his own movements without seeing himself, he feels how “all of him” is in the room and how he is “in his body.” He thinks he creates a “more holistic expression” when he is moving from “within” than if he sees himself performing the movement from the outside. According to Anders, the “gaze” fails to focus on the whole body at the same time. When he is to perform the standard movements, the other's gaze is directed at various body parts, such as the face, arms or legs. This means, according to Anders, that the outside view can never see the “whole” that Anders experiences when he creates dance.

When the students create dance alone, in this final part of the project, they use music as both inspiration and structure. Thomas (TI1) lets the music “control” which standard movements he chooses for his dance, but he also includes more unknown movements that “sit in the body” from having danced at various nightclubs. In addition, he “copies” movements he has learnt from other students, who he believes also “copy” movements from him when they create their own dances. “Being inspired by each other” feels good, says Thomas. In this way, Thomas includes standard movements, movements he himself has experienced, movements he has seen his friend do, and movements they have created together. When the movements are to be put together into a dance, Thomas first lays out a “basic plan” for where to do the various chosen movements before going on to explore how these movements can be further explored dynamically, “slower” or “faster.”

Through activating and exploring their internalized movements, the students' lived presence of the past opens a procedural field of possibilities, affordances and probabilities when they are creating dance alone (Fuchs, 2017). When the students chose set movements, they became aware of what the movements looked like “from the outside.” When they performed movements from their own body memory, they became aware of how they experienced the movement “from within,” with the whole body. The combining of own and set dance movements can, according to concepts taken from Merleau-Ponty (2012), be understood as the students creating dances based on both their lived body and their objective body.

Although the students are creating the dances by listening to and structuring the movements to the experience of the music, they are also aware of movements they have learnt and created together with their fellow students. At the beginning of the project, they were afraid of what their fellow students would think about the way they moved. Now, however, the students are inspired to explore the movements made by the other students and to try and further explore these with their own bodies. At this point, through the concepts from Fuchs (2016), the students are using their intercorporeal memory of moving with peer students in their dances, and in this way they are incorporating the other students' body memories as part of their new movement patterns. In sum, through their lived space, as Fuchs (2017) describes it, the students' creation of dance in the classroom by way of this bodily approach includes their previous experiences of creating dance: in various situations, with several students, in a variety of movements and within a wide range of possible structures. How the students experience the exploration of this way of creating dance will be shown in the final theme, which follows.

### New Ways of Moving and Understanding the Creation of Dance

Creating dance is something Charlotte (CHR2) eventually thinks is “very fun,” “exciting,” and “easy,” because she can do whatever she “wants to do,” without feeling she is performing a “wrong” or “bad” movement in accordance to the predetermined set movement rules of others. Now, like the other students, she has become more comfortable with creating dance based on her own movement experiences, interests and possibilities. This confidence encourages the students to explore new ways of moving. Vidar (VI2) assumes he must “explore new movements” by moving. If he “analyses” the movements before he performs them, he “ends up” with the “safe,” predetermined movements he already knows. If he is to be able to move in new ways, “the body and the mind must go at the same time.” Instead, by lying down on the floor and starting to move, he thinks he can manage to create “completely new” movements he has not “come up with before” and which only “go by themselves.” For both Vidar (VI2) and Anders (AI2), creating new movements is thus an interplay between moving and thinking. Every time Vidar makes a movement, it makes him think of new movement possibilities. Thus, for Vidar, creating dance is not about “whims” but about “conscious choices for further movements.” This coincides with the thought of Anders, who experiences the creation of new movements by being “in the body” and at the same time thinking about which movements are possible as the next step in the dance. For Charlotte (CHI2), the body plays an “important role” in creating her dances because she experiences the body as a medium from which she can select movements and as the expression of these movements. In this way, she makes a distinction between her experience of her body in movement exploration, where the body is something she *has* and which *is*, and her experience of performing the dance, where the body is the object of her dance. Many of the students experience their body in the same way. Anders (AI2) says that “the body is you, and the more you use the body, the more you use yourself,” and when he creates new movements, he has the experience of “using more of [myself]”. For the students, “using oneself” offers an opportunity to “put something of yourself into the dance” (SI1), where their final dance is about expressing themselves for others.

In this final stage of the project, the students created dances without having “a set plan” – such as stories or music – for how to create and how the final dance should look. For Anders (AR2), this open structure for creating dance gives him the opportunity to be “himself” and to “give the body meaning”. He also finds that creating dance “from [his] body” gives him “something more” than “competitive activities” because the experience of dancing with new movements makes dance a “unique experience” that is in line with his “identity” and his “view of human movement and the body.” For Jens (JI1), this “unique experience” is a “journey” that gives him the opportunity to explore the “limits” of what movements his body can, and will, perform. This bodily way of creating new movements based on movement explorations gives him the opportunity to explore movements his “imagination” and “creativity” had previously erected “barriers” against.

When the students began exploring new movements based on their previous experience of creating dance alone, then with a student they trust and then in groups, expressions such as “give the body meaning,” “unique experience,” “identity,” “to use oneself,” and “to show oneself” emerged. These experiences are in line with what Nilges (2004) refers to as the expressive and the experimental dimensions of creating dance. According to the studies of Cone (2009) and Mattsson (2016), creating dance can be a way of expressing oneself. The point of departure for one to enable oneself to express “themselves” is what Fuchs (2017) refers to as the lived body and what Nilges (2004) refers to as the sensory dimension of creating dance. By sensing their own body when moving, a person decides what kind of movement they do – or don't – want to include in their dance, based on what the movement makes them think of while they are moving. By contrast, if a person starts to think during the creative process about how to move, they will end up performing movements they already know. When the students moved without first “analyzing” their movements, they found that “completely new” movements developed “by themselves,” which went on to create new cognitive and bodily ideas about how they could continue to be in the creative process. In this way, cognitive strategies and implicit movement dispositions worked together, but the creative process starts with being in movement. This interplay between body, movement and thought can further be seen in the light of Fuchs' (2016) concept of embodied affectivity, where affective and emotive experiences influence how one feels and thinks while creating dance. This operative intentionality of the body in connection with the environment opens a procedural field of possibilities, affordances and probabilities where the students' bodily being-toward-the-world (Merleau-Ponty, 2012) is made visible through their dance.

## Conclusion

In this conclusion, I use the concepts *embodied inter/affectivity, body memory, social musicality*, and *participatory sense-making* (De Jaegher and Di Paolo, 2007; Fuchs, 2016, 2017) to discuss the second research question (how were these experiences facilitated by bodily learning that was initiated by body memories?).

In creating dance, the student teachers attune themselves to one another's rhythms, dynamics and patterns of interactions, while exploring, transforming and creating movements through their *embodied affective* and *embodied interaffective* interaction with the environment and the other student teachers (Fuchs, 2016, 2017). The socially interactive teaching situation trigger the student teachers' bodily and interbodily resonances that further create affective and emotive experiences when the student teachers create dances alone, and together with the other students (Fuchs, 2016, 2017). These bodily experiences form the existential basis of the class culture and the learning environment in creative dance.

This intersubjective and intercorporeal approach to creative dance has given the students time to reflect on their movements, their bodily and interbodily resonances and thoughts while creating dance, which has allowed them to become aware of how their body memories influence their understand of creating dance, their bodies and one another. Based on the results of this study, bodily learning through creative dance includes re-activating the students' subjective, dyadic, intercorporeal and collective body memories, exploring their bodily and interbodily resonances while moving and dancing, creating new body memories through creating dances and reflection on their experiences. This has led toward an understanding of how bodily learning in creative dance can be explored through both embodied and language-based ways of knowing (Chappell, 2007) as long as the students are given the opportunity to explore movements that they know, are interested in and are curious about. In line with Oppici et al. (2020), the educational potential of bodily learning through the creation of dance can be seen in relation to the affordances the students perceive and utilize within their learning culture in PETE. This require a PETE education that understand creative dance as a cultural practice, performed and shaped prereflectively within the embodied and implicit knowing in the collective memory of moving and dancing within a PETE context.

The eight descriptemes: “dreading the lessons,” “creating dance based on own movement experiences,” “anxiety about not fitting in,” “the fear of, and the desire to, express emotions,” “confidence to create dance,” “sharing and adjusting to other's movement experiences,” “exploring own movement experiences, movement opportunities and movement interests,” and “new ways of moving and understanding the creation of dance,” illuminate how the student teachers' develop a prereflective, practical knowledge of how to get along with each other while creating dances over time. Through the creation of new affective-interactive schemas, the student teachers develop their social musicality that enables the formation and tradition of new collective patterns of interaction in creative dance in a PETE context. These results shows that creating dance in a PETE context, in line with current research in dance, is an ongoing intersubjective, intercorporeal and interaffective process that becomes a part of the student teachers' embodied personality structure (Fuchs, 2017).

The eight descriptemes also present how the student teachers' social musicality is experienced both as an individual activity and as a joint process of sense-making (De Jaegher and Di Paolo, 2007). This ongoing individual and participatory sense-making process can further be understood, in line with Lobel (2021), as a reciprocal dependency between culture and self, where the students' mutual incorporation and resonance occur and evolve on an existential level over time. By way of this process, the students develop a sense of confidence and trust in each other, as Rustad and Fjogstad Langnes (2019) also show, which creates a sense of belonging (Amado et al., 2020). Through this participatory sense-making process, the students develop a practical sense of how to move and dance that is now part of their embodied personality structure (Fuchs, 2016, 2017).

These findings reveal a need for further research into how body memories may be relevant to understanding the learning of bodily learning through creative dance. Further research on how creating dance can facilitate bodily learning among and between groups of students and maybe how creating dance can foster an inclusive and supporting learning environment are welcomed. I also suggest further research be undertaken into how student teachers, teachers and teacher educators in PETE become cognisant of the implications of creative dance as a bodily learning process, as a counterweight to the more competitive focus in PETE. This research will be relevant in order to further discuss the value of creating dance as a subject in PE and PETE, and to initiate a debate on how awareness of intersubjective, intercorporeal and interaffective experiences may be relevant to understanding learning in PE and PETE in general.

## Data Availability Statement

The original contributions presented in the study are included in the article/supplementary material, further inquiries can be directed to the corresponding author/s.

## Ethics Statement

The studies involving human participants were reviewed and approved by the Norwegian Centre for Research Data. The participants provided their written informed consent to participate in this study.

## Author Contributions

The author confirms being the sole contributor of this work and has approved it for publication.

## Funding

This work was funded by the strategical research program “You will become a teacher…” at the University of South-Eastern Norway.

## Conflict of Interest

The author declares that the research was conducted in the absence of any commercial or financial relationships that could be construed as a potential conflict of interest.

## Publisher's Note

All claims expressed in this article are solely those of the authors and do not necessarily represent those of their affiliated organizations, or those of the publisher, the editors and the reviewers. Any product that may be evaluated in this article, or claim that may be made by its manufacturer, is not guaranteed or endorsed by the publisher.

## Footnotes

^1^This approach to creating dance can for example be seen in the books *Dance Composition* (Smith-Autard, 2010), *Dance Composition Basics* (Sofras, 2006) and *With the Body as Material: About dance in practice* (Ravn, 2014).

## References

[B1] Amado D. Molero P. Del Villar F. Tapia-Serrano M. Á. Sánchez-Miguel P. A. (2020). Implementing a teacher-focused intervention in physical education to increase pupils' motivation towards dance at school. Sustainability 12:4550. 10.3390/su12114550

[B2] Anttila E. (2006). Dance and dialogue: partnership and beyond, in Finnish Dance Research at the Crossroads: Practical and Theoretical Challenges, eds Pakkanen P. K. Sarje A. (Helsinki: Arts Council of Finland).

[B3] Bae B. (2016). Å se barn som subjekt - noen konsekvenser for pedagogisk arbeid I barnehage. Available online at: https://www.regjeringen.no/no/tema/familieog-barn/barnehager/artikler/a-se-barn-som-subjekt—noen-konsekvense/id440489/ (accessed October 1, 2007).

[B4] Booth S. Woollacott L. (2015). The Scholarship of Teaching and Learning: On Its Constitution and Transformative Potential. Stellenbosch: Sun Press. 10.18820/9780992236052

[B5] Boyer E. L. (1990). Scholarship Reconsidered: Priorities of the Professoriate. San Francisco, CA: Jossey-Bass.

[B6] Bresler L. (2006). Toward connectedness: aesthetically based research and its ethical implications. Stud. Art Educ. J. Issues Res. 48, 52–69. 10.1080/00393541.2006.11650499

[B7] Chappell K. (2007). Creativity in primary level dance education: moving beyond assumption. Res. Dance Educ. 8, 27–52. 10.1080/14647890701272795

[B8] Chen W. (2000). Self-assessing expertise in creative dance teaching from constructivist perspectives. Teach. Element. Phys. Educ. 11, 4–6.

[B9] Chen W. Cone T. (2003). Links between children's use of critical thinking and an expert teacher's teaching in creative dance. J. Teach. Phys. Educ. 22, 169–185. 10.1123/jtpe.22.2.169

[B10] Cleland Donnelly F. E. Millar V. F. (2019). Moving green, going green: an interdisciplinary creative dance experience. J. Phys. Educ. Recreat. Dance 90, 20–33. 10.1080/07303084.2019.1637304

[B11] Cone T. (2009). Following their lead. Supporting children's ideas for creating dances. J. Dance Educ. 9, 81–89. 10.1080/15290824.2009.10387390

[B12] Daly A. (1994). Isadora duncan's dance theory. Dance Res. J. 26, 24–31. 10.2307/1477914

[B13] De Jaegher H. Di Paolo E. (2007). Participatory sense-making: an enactive approach to social cognition. Phenomenol. Cognit. Scie. 6, 485–507. 10.1007/s11097-007-9076-9

[B14] Engel L. (2004). På sporet af bevgelse - imagination og kreative processer. Available online at: https://idrottsforum.org/articles/engel/engel.html

[B15] Foster S. L. (2011). Choreographing Empathy. Kinesthesia in Performance. New York, NY: Routledge. 10.4324/9780203840702

[B16] Fraleigh S. (2004). Dancing Identity. Metaphysics in Motion. Pittsburgh, PA: University of Pittsburgh Press. 10.2307/j.ctt5hjq68

[B17] Froese T. Fuchs T. (2012). The extended body: a case study in the neurophenomenology of social interaction. Phenomenol. Cognit. Sci. 11, 205–235. 10.1007/s11097-012-9254-2

[B18] Fuchs T. (2016). Intercorporeality and interaffectivity. Phenomenol. Mind 11, 194–209. 10.1093/acprof:oso/9780190210465.003.0001

[B19] Fuchs T. (2017). Collective body memories, in Embodiment, Enaction, and Culture. Investigating the Constitution of the Shared World, eds Durt C. Fuchs T. Tewes C. (Cambridge, CA: MIT Press). 10.7551/mitpress/9780262035552.003.0018

[B20] Fuchs T. De Jaegher H. (2009). Enactive intersubjectivity: participatory sense-making and mutual incorporation. Phenomenol. Cognit. Sci. 8, 465–486. 10.1007/s11097-009-9136-4

[B21] Hämäläinen S. (2006). Two different approaches in teaching choreography - effects on movement exploration and composition, in Finnish Dance Research at the Crossroads: Practical and Theoretical Challenges, eds Pakkanen P. K. Sarje A. (Helsinki: Arts Council of Finland).

[B22] Hawkins A. M. (1991). Moving From Within. A New Method for Dance Making. Chicago, CA: A Cappella Books.

[B23] H'Doubler M. N. (1998). Dance. A Creative Art Experience. Wisconsin: University of Wisconsin Press.

[B24] Keun L. L. Hint P. (2006). Creative dance: Singapore children's creative thinking and problem-solving responses. Res. Dance Educ. 7, 35–65. 10.1080/14617890600610661

[B25] Kvale S. Brinkmann S. (2009). Det kvalitative forskningsintervju. Oslo: Gyldendal.

[B26] Laban R. (1948). Modern Eductional Dance. London: MacDonald and Evans.

[B27] Laban R. (1966). Choreutics. London: Macdonald and Evans.

[B28] Laban R. Lawrence C. F. (1947). Effort. London: Macdonald and Evans.

[B29] Lara-Aparicio M. Mayorga-Vega D. López-Fernández I. (2021). Expressive movement & creative dance practice in times of quarantine: the #vidlop movement. Movimento 27, 1–19. 10.22456/1982-8918.105802

[B30] Larsson H. Karlefors I. (2015). Physical education cultures in sweden: fitness, sports, dancing … learning? Sport Educ. Soc. 20, 573–587. 10.1080/13573322.2014.979143

[B31] Lobel E. E. (2021). A model for integrating social and creative dance forms in a dance education course. J. Dance Educ. 21, 44–47. 10.1080/15290824.2020.1732984

[B32] Mattsson T. (2016). Expressiva dansuppdrag: utmanande läruppgifter i ämnet idrott och hälsa. PhD diss. Malmö høgskola.

[B33] Merleau-Ponty M. (2012). Phenomenology of Perception. London: Routledge. 10.4324/9780203720714

[B34] Minton S. (2007). Middle school choreography class: two parallel but different worlds. Res. Dance Educ. 8, 103–121. 10.1080/14647890701706081

[B35] Morin F. (2008). Composing dances with children an instructional model. J. Phys. Educ. Recreat. Dance 72, 43–49. 10.1080/07303084.2001.10605770

[B36] Neal N. D. (1981). Sports movement in dance composition. J. Phys. Educ. Recreat. 52, 42–49.

[B37] Neville R. D. Makopoulou K. (2021). Effect of a six-week dance-based physical education intervention on primary school children's creativity: a pilot study. Eur. Phys. Educ. Rev. 27, 203–220. 10.1177/1356336X20939586

[B38] Oppici L. Frith E. Rudd J. (2020). A perspective on implementing movement sonification to influence movement (and eventually cognitive) creativity. Front. Psychol. 11:2233. 10.3389/fpsyg.2020.02233 33071849 PMC7531231

[B39] Ørbæk T. (2018). Å* skape dans i kroppsøvingslærerutdanning - studenters erfaringer. (Doktorgradsavhandling)*. Oslo: Norges idrettshøgskole.

[B40] Ørbæk T. (2021). Å utvikle lærerstudenters profesjonskunnskap gjennom kroppslig læring, in Kroppslig læring. Perspektiver og praksiser, eds Østern T. P. Bjerke Ø. Sørum A. G. Engelsrud G. (Oslo: Universitetsforlaget).

[B41] Ørbæk T. Engelsrud G. (2019). Skapende dans i en skolekontekst - et forskningsfelt? Dance Articul. Special Issue Bodily Learn. 5, 4–26. 10.18862/ps.2019.502.2

[B42] Østern A.-L. Nødtvedt Knudsen K. (2019). Performative Approaches in Arts Education: Artful Teaching, Learning and Research (1st Edition). London: Routledge. 10.4324/9780429444159

[B43] Østern T. P. (2009). Meaning-Making in the Dance Laboratory : Exploring Dance Improvisation With Differently Bodied Dancers (Doctoral thesis). Acta Scenica.

[B44] Østern T. P. (2017). Å forske med kunsten som metodologisk praksis med aesthesis som mandate. J. Res. Arts Sports Educ. 1, 7–27. 10.23865/jased.v1.982

[B45] Østern T. P. Bjerke Ø. Engelsrud G. Grut Sørum A. (2021). Kroppslig læring. Perspektiver og praksiser. Oslo: Universitetsforlaget.

[B46] Østern T. P. Dahl T Strømme, A. Aagaard Petersen J. Østern A.-L. Selander S. (2019). Dybde/læring - en flerfaglig, relasjonell og skapende tilnærming. Oslo: Universitetsforlaget.

[B47] Østern T. P. Engelsrud G. (2014). Læreren-som-kropp, in Dramaturgi i didaktisk kontekst, ed Østern A. L. (Bergen: Fagbokforlaget).

[B48] Payne H. Costas B. (2020). Creative dance as experiential learning in state primary education: the potential benefits for children. J. Experi. Educ. 44, 277–292. 10.1177/1053825920968587

[B49] Petitmengin C. Remillieux A. Valenzuela-Moguillansky C. (2018). Discovering the structures of lived experience. Towards a micro-phenomenological analysis method. Phenomenol. Cognit. Sci. 18, 691–730. 10.1007/s11097-018-9597-4

[B50] Ravn S. (2009). Sensing Movement, Living Spaces. An Investigation of Movement Based on the the Lived Experience of 13 Professional Dancers. Saarbrücken: VDM Verlag.

[B51] Ravn S. (2014). Med kroppen som materiale: om dans i praksis. Odense: Syddansk Universitetsforlag.

[B52] Reason M. Reynolds D. (2012). Kinesthetic Empathy in Creative and Cultural Practices. Bristol: NBN International.

[B53] Rustad H. (2013). Dans etter egen pipe? En analyse av danseimprovisasjon og kontaktimprovisasjon - som tradisjon, fortolkning og levd erfaring. PhD diss., Norges idrettshøgskole.

[B54] Rustad H. Fjogstad Langnes T. (2019). Flashmob i Lærerutdanninger - studenters erfaringer med å skape dans sammen. J. Res. Arts Sports Educ. 3, 46–61. 10.23865/jased.v3.1448

[B55] Sheets-Johnstone M. (2009). The Corporeal Turn. An Interdisciplinary Reader. Exeter: Imprint.

[B56] Sklar D. (2001). Dancing with the Virgin. Body and Faith in the Fiesta of Tortugas, New Mexico. London: University of Carlifornia Press.

[B57] Smith-Autard J. M. (2010). Dance Composition: A Practical Guide to Success in Dance Making. London: A&C Black.

[B58] Sofras P. A. (2006). Dance Composition Basics: Capturing the Choreographer's Craft. Champaign, IL: Human Kinetics.

[B59] Steinberg C. Steinberg F. (2016). Importance of students' views and the role of self-esteem in lessons of creative dance in physical education. Res. Dance Educ. 17, 189–203. 10.1080/14647893.2016.1208646

[B60] Torrents C. Castañer M. Dinušová M. Anguera T. (2012). Dance divergently in physical education: teaching using open-ended questions, metaphors, and models. Res. Dance Educ. 14, 104–119. 10.1080/14647893.2012.712100

[B61] Varea V. González-Calvo G. Martínez-Álvarez L. (2018). Exploring touch in physical education practicum in a touchy latin culture. Societies 8:54. 10.3390/soc8030054

[B62] Velten Rothmund I. (2019). Å* gjøre dansen til sin: Bachelorstudenters levde erfaringer i moderne- og samtidsdans*. PhD diss., StockholmsUniversitet.

[B63] Vertinsky P. (2010). From physical educators to mothers of the dance: Margaret H'Doubler and Martha Hill. Int. J. History Sport 27, 1113–1132. 10.1080/09523361003695785

